# Diagnostic Radiology Fellowship Applicant Selection Criteria: A Survey of Program Directors

**DOI:** 10.7759/cureus.38409

**Published:** 2023-05-01

**Authors:** Rajbir S Pannu, Nicholas Mills, Alexander Tsibulski, George Pappas

**Affiliations:** 1 Department of Diagnostic Radiology, Trinity Health Oakland Hospital, Pontiac, USA

**Keywords:** program director survey, radiology fellowship, medical education, fellowship training, diagnostic radiology

## Abstract

Introduction

In this study, we aimed to investigate the importance of various characteristics used by diagnostic radiology subspecialty fellowship programs when selecting candidates for their fellowship programs.

Methods

An online survey was sent to program directors from multiple radiology fellowship programs in the United States. Multiple questions were asked, including applicant gender, personal statement, interview performance, research experience, United States Medical Licensing Exam (USMLE) performance, and residency program attended. Survey recipients were asked to rank these characteristics from least to most important.

Results

A total of 119 responses were collected (response rate of 26.2%), with a relatively balanced representation of the different subspecialties. An aggregate analysis of all the subspecialties showed interview performance, letters of recommendation, and residency program attended as the three most important factors considered by the program directors when selecting a candidate for their fellowship program. In contrast, the three least important factors were gender, prior subspecialty training, and minority status. The applicant's visa status, personal statement, and USMLE Step 3 performance were the only statistically significant (p<0.05) differences between the subspecialties. The women's and body imaging subspecialties rated the personal statement more important than musculoskeletal imaging and neuroradiology. Cardiothoracic and musculoskeletal imaging rated the applicant's visa status more important than neuroradiology. Women's and cardiothoracic imaging rated USMLE Step 3 performance as more important than musculoskeletal imaging.

Conclusion

Selecting the ideal candidate for a fellowship in radiology can be difficult. Our findings outline which applicant characteristics program directors believe are the most and least important when selecting an ideal candidate. For instance, personal characteristics such as interview performance and letters of recommendation are more valued than extracurricular activities such as research experience. Identifying these characteristics provides a better understanding of the fellowship application process and may guide future applicants.

## Introduction

Diagnostic radiology training in the United States includes a four-year program preceded by a one-year prerequisite program. After completing five postgraduate years of training, the physician can pursue subsequent subspecialty fellowship training. The vast majority of residents pursue fellowship training for many reasons, some of which include educational interests and preferred employment. A survey conducted in 2009 found that 93.4% of residents pursued fellowship training after completing residency, whereas 80.1% of residents pursued fellowship training in 1999 [1]. Additionally, fellowship-trained radiologists often have higher salaries than their non-fellowship-trained counterparts in both academic and private-practice settings [2]. Typically, residents must decide which subspecialty to choose early in their residency as most applications must be sent early in their third year. There is no common application platform for the subspecialties; however, subspecialties such as neuroradiology, breast imaging, and musculoskeletal radiology use the National Resident Matching Program, which can make it easier for those applying to these subspecialties [3]. This can add another layer of complexity to the application process. The study investigates which characteristics are deemed the most and least important by program directors from various diagnostic radiology subspecialty fellowship programs when selecting a fellowship candidate.

## Materials and methods

Institutional review board approval for this study was obtained from Saint Joseph Mercy, Oakland (approval #2021-059-sjmo).

An online survey was conducted on radiology fellowship program directors in the United States between January 2022 and April 2022 regarding the criteria for selecting a fellowship candidate. The survey was administered using the online survey product SurveyMonkey.com LLC. A total of six different radiology subspecialties were surveyed: abdominal/body/cross-sectional imaging, breast/women's imaging, cardiothoracic imaging, musculoskeletal imaging, neuroradiology imaging, and pediatric imaging. The survey included a general yes/no consent question and a multiple choice question regarding the program director's subspecialty. The subsequent 22 questions were grouped into four categories of demographics, personal characteristics, extracurricular activities, and educational accomplishments (Table 1). A standard 10-point Likert scale was used to rank each applicant's characteristics, with 10 being the most important and one being the least important characteristic.

**Table 1:** Primary questionnaire which was associated with a 10-point Likert scale. USMLE: United States Medical Licensing Exam; US MD: United States Doctor of Medicine; US DO: United States Doctor of Osteopathic Medicine; GPA: Grade Point Average.

General Grouped Category	Applicant Characteristics
Demographics	Geographic ties to the region of the program
	Visa status (J1/H1B/Green Card/Citizenship)
	Degree type (US MD, US DO, or International Graduate)
	Prior Subspecialty Training
	Gender
	Minority Status
Personal Characteristics	Letters of recommendation
	Interview performance
	Personal statement
	Residency completed at the same institution
	External candidate evaluated during prior interaction
Extracurricular Activities	Research experience
	Hobbies
	Additional degrees
	Leadership experience
	Teaching experience
Educational Accomplishments	USMLE* Step 1 and 2 performances
	USMLE* Step 3 performance
	Medical school attended
	Medical school performance (i.e., GPA, clinical honors, etc.)
	Residency program attended
	In-service exam performance

The Society of Abdominal Radiology, Society of Breast Imaging, Society of Thoracic Radiology, Society of Skeletal Radiology, American Society of Neuroradiology, and the Society of Pediatric Radiology websites were browsed to obtain the institutions which offered the corresponding fellowship programs. Subsequently, the individual websites for the fellowship programs were visited, and the program directors' names and email addresses were obtained. The program coordinators' information was obtained for those programs that did not offer the program directors' email addresses. The program directors were sent a standardized email invitation to participate in the study, which included a brief description of the survey, the privacy policy of the data collected, and information regarding the principal investigators. When the program director's email was unavailable, a similar email was sent to the program coordinators, which included the options of forwarding the survey hyperlink and invitation to the corresponding program director's email address or requesting the program director's email address. In all instances, the survey recipients were informed that the responses were anonymous. A total of 455 programs were sent the survey via email. These included 79 abdominal/body/cross-sectional imaging, 91 breast/women's imaging, 57 cardiothoracic imaging, 89 musculoskeletal imaging, 90 neuroradiology imaging, and 49 pediatric imaging programs. The survey data were exported for statistical analysis using SPSS statistics software version 25. Statistical significance was assessed using analysis of variance. A p-value of less than 0.05 was considered to represent a statistically significant association. 

## Results

**Figure 1:**
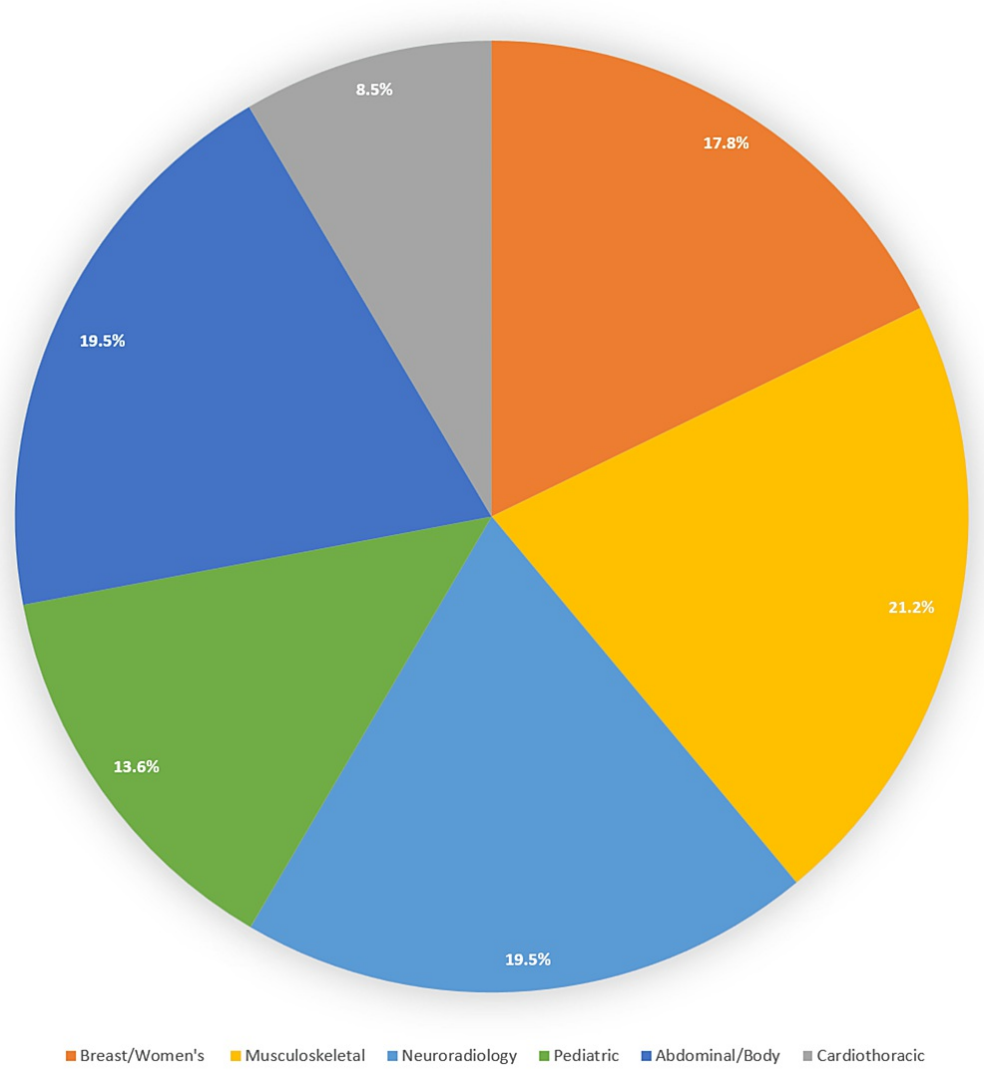
Subspecialties of the respondents. Color-coordinated pie chart demonstrating the percentage distribution of the subspecialties.

One hundred nineteen responses were collected from the invitation, corresponding to an overall response rate of 26.2% (119 of 455). It should be noted that nine invitations were confirmed to have been automatically declined by the recipient's email platform or bounced; furthermore, it is uncertain how many of the email addresses were inactive. Therefore, the actual response rate to the survey may have been higher. Additionally, one of the respondents failed to identify their subspeciality and was excluded from the analysis. A relatively balanced response was collected from different subspecialty program directors (Figure 1), including 21 breast/women's imaging, 25 musculoskeletal imaging, 23 neuroradiology imaging, 16 pediatric imaging, 23 abdominal/body/cross-sectional imaging, and 10 cardiothoracic imaging programs.

**Figure 2:**
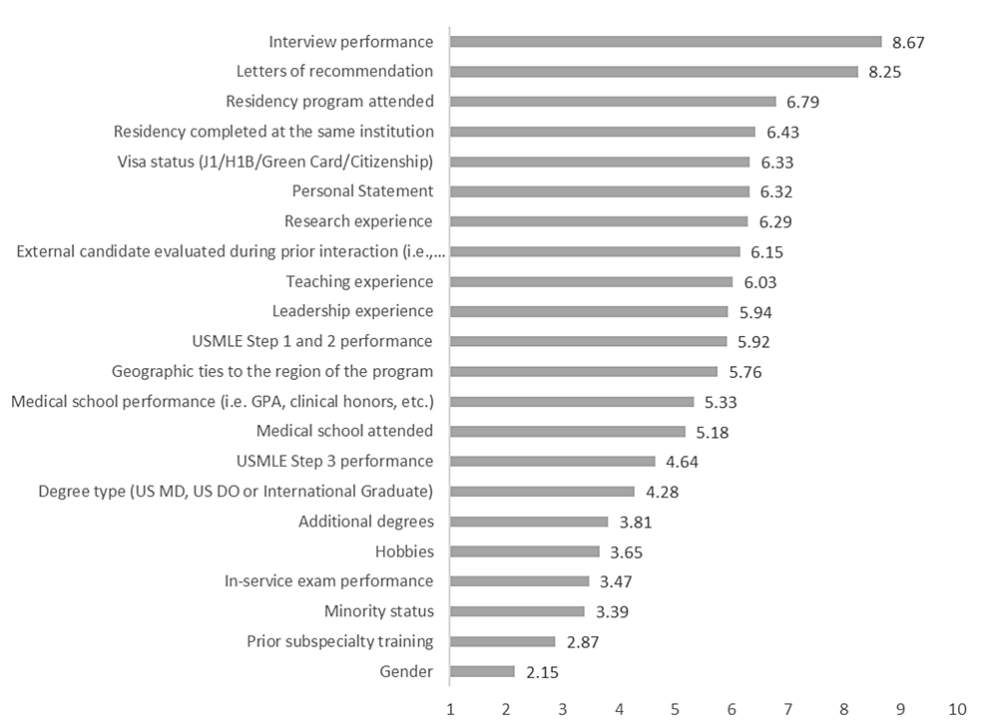
The overall ranking of the various applicant characteristics. The clustered bar graph ranking the applicants' characteristics from the most important at the top and least important at the bottom. Numerical values to the right of the bars indicate the mean score from the Likert scale. USMLE: United States Medical Licensing Exam; US MD: United States Doctor of Medicine; US DO: United States Doctor of Osteopathic Medicine; GPA: Grade Point Average.

An aggregate analysis of all the subspecialties showed interview performance, letters of recommendation, and residency program attended as the three most important factors considered by the program directors when selecting a candidate for their fellowship program. The three least important factors are gender, prior subspecialty training, and minority status. Figure 2 summarizes the overall ranking of the various applicant characteristics.

**Figure 3:**
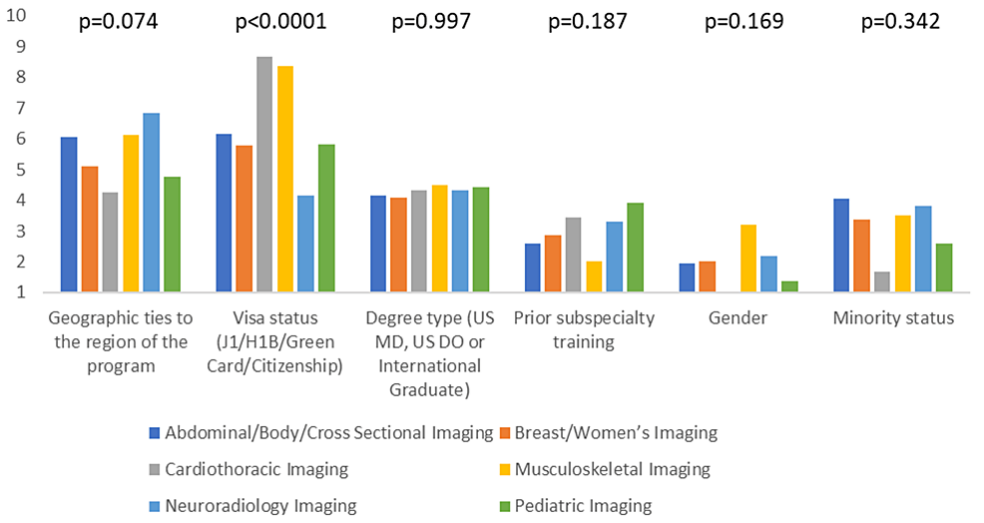
The clustered column graph compares the rankings of different applicants' demographics amongst the different subspecialties. P-values are included above the corresponding columns. Columns are based on the mean score from the Likert scale. US MD: United States Doctor of Medicine; US DO: United States Doctor of Osteopathic Medicine.

**Figure 4:**
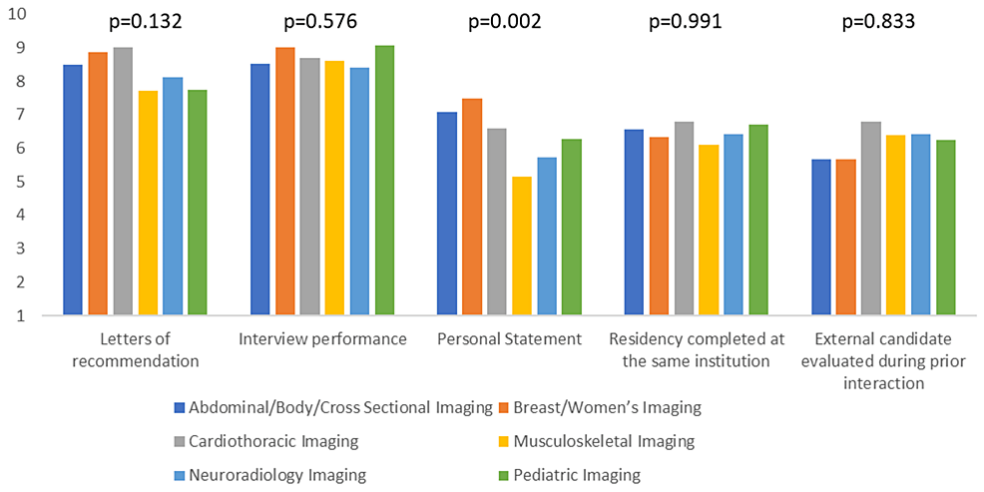
The clustered column graph compares the rankings of applicants' personal characteristics amongst the different subspecialties. P-values are included above the corresponding columns. Columns are based on the mean score from the Likert scale.

**Figure 5:**
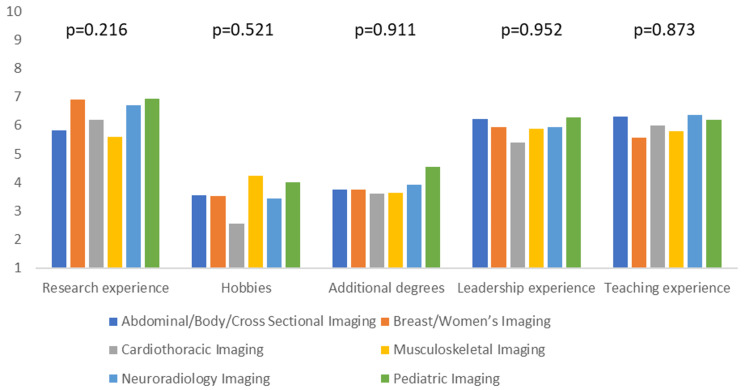
The clustered column graph compares the rankings of applicants' extracurricular activities amongst the different subspecialties. P-values are included above the corresponding columns. Columns are based on the mean score from the Likert scale.

**Figure 6:**
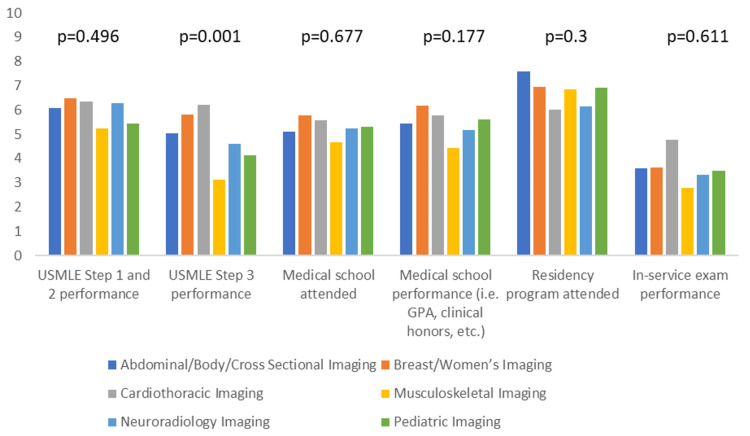
The clustered column graph compares the rankings of applicants' educational accomplishments amongst the different subspecialties. P-values are included above the corresponding columns. Columns are based on the mean score from the Likert scale. USMLE: United States Medical Licensing Exam; GPA: Grade Point Average.

Additionally, we assessed if there is any difference amongst the different subspecialties regarding the most and least important applicant characteristics. The only statistically significant differences were observed with visa status, personal statement, and USMLE step 3 performance (p-value <0.05). Cardiothoracic and musculoskeletal imaging ranked visa status significantly higher than other specialties (mean score of 8.7 and 8.4, respectively), whereas neuroradiology ranked it the lowest (mean score of 4.1). Breast/Women’s imaging and abdominal/body/cross-sectional imaging ranked the personal statement significantly higher than other specialties (mean score of 7.5 and 7.1, respectively). In contrast, musculoskeletal and neuroradiology imaging ranked it the lowest (mean score of 5.2 and 5.7, respectively). There was no significant variation amongst the subspecialties in the general category of extracurricular activities. Cardiothoracic and breast/women’s imaging ranked the USMLE step 3 performance significantly higher than the other subspecialties (mean score of 6.2 and 5.8), whereas musculoskeletal imaging ranked it the lowest (mean score of 3.1). Figures 3-6 summarize the data for comparing applicant characteristics amongst the different subspecialties.

## Discussion

The fellowship application can be daunting for a resident; therefore, early preparation is essential in obtaining a preferred spot. Often, residents have to select a subspecialty early in their residency and subsequently work on building their curriculum vitae. Research, teaching, and leadership experience require years of preparation and planning. Interestingly, this survey shows that the two most important applicant qualities are personal characteristics: interview performance and letters of recommendation. These findings are similar to a study conducted in 2012 demonstrating personal interviews, letters of recommendation, and personality as the most important subjective factors for selecting a fellow [4]. The aforementioned study demonstrated residency performance and the prestige or quality of the applicant's residency program as the most important objective factors. Similarly, this survey showed that the third most important characteristic was the residency program attended. A similar survey in 2020, specifically on neuroradiology fellowships, showed similar findings [5]. The applicant's residency program attended, personality traits assessed during the interview process, and previous experience in the fellowship's geographic region were ranked high by the program directors. However, research experience was ranked as the third most important criterion in the selection process, whereas our survey ranked research experience as the seventh most important factor. Residency completed at the same institution, visa status, personal statement, research experience, external candidate evaluated during prior interaction, teaching experience, leadership experience, USMLE step 1 and 2 performance, geographic ties to the region of the program, and medical school attended were relatively similar in performance ranging from an average score of 6.4 to 5.2, respectively. The two least important characteristics include the applicant's gender and prior subspeciality training. Interestingly, minority status held greater importance than prior subspecialty training (average score of 3.4 versus 2.9).

To our knowledge, no other survey has been conducted to determine if any applicant characteristics differ between subspecialties. The applicant's personal statement was considered more important in women's imaging and body imaging and least important in musculoskeletal imaging. The reason for this is uncertain and further research may be needed. Although cardiothoracic and musculoskeletal imaging value visa status more than neuroradiology, it is unclear whether this had a detrimental or positive impact on the applicant's success. The study is limited with regard to sample size. Although nearly all the subspecialty fellowship programs were contacted, the response rate was lower than anticipated. Multiple reasons could explain this finding. For example, a web-based survey was utilized, which required email as the primary form of communication. Web-based surveys are easy to conduct, so the recipients may have received multiple surveys from various sources and perhaps chose to ignore a few of these surveys. Additionally, we chose a sample time spanning from January to April, which coincides with the fellowship cycle. Program directors may have been preoccupied with conducting interviews with limited time to complete the survey. It is uncertain to what extent, if any, this limited sample size affected the results. Nevertheless, our response rate is comparable to that obtained by a similar survey conducted in 2012 [4].

## Conclusions

Selecting the ideal candidate for a fellowship in radiology can be difficult. As a candidate, it is essential to know early on what the fellowship program is looking for so that the application can be tailored for success. Nonetheless, specific fixed characteristics, such as the residency program, are important and must be considered by the applicants.
